# Brown Seaweed Fucoidan in Cancer: Implications in Metastasis and Drug Resistance

**DOI:** 10.3390/md18050232

**Published:** 2020-04-28

**Authors:** María Elena Reyes, Ismael Riquelme, Tomás Salvo, Louise Zanella, Pablo Letelier, Priscilla Brebi

**Affiliations:** 1 Laboratory of Integrative Biology (LIBi), Center of Excellence in Translational Medicine- Scientific and Technological Bioresource Nucleus (CEMT-BIOREN), Universidad de La Frontera, Temuco 4710296, Chile; 2 Instituto de Ciencias Biomédicas, Facultad de Ciencias de la Salud, Universidad Autónoma de Chile, Temuco 4810101, Chile; 3 Precision Health Research Laboratory, Departamento de Procesos Diagnósticos y Evaluación, Facultad Ciencias de la Salud, Universidad Católica de Temuco, Temuco 4813302, Chile

**Keywords:** fucoidan, cancer, metastasis, epithelial mesenchymal transition, nanoparticles

## Abstract

Fucoidans are sulphated polysaccharides that can be obtained from brown seaweed and marine invertebrates. They have anti-cancer properties, through their targeting of several signaling pathways and molecular mechanisms within malignant cells. This review describes the chemical structure diversity of fucoidans and their similarity with other molecules such as glycosaminoglycan, which enable them to participation in diverse biological processes. Furthermore, this review summarizes their influence on the development of metastasis and drug resistance, which are the main obstacles to cure cancer. Finally, this article discusses how fucoidans have been used in clinical trials to evaluate their potential synergy with other anti-cancer therapies.

## 1. Introduction

Fucans are a family of polymeric molecules composed by a simple and long structure based on fucose and sulphate. Fucoidans are a subgroup within the fucan family, consisting of polysaccharides that are composed of sulphated l-fucose (6-deoxy-l-galactose) produced mainly by brown algae and, to a lesser extent, by marine invertebrates [1].

Due to the structural similarity between fucoidans and certain sulphated polysaccharides from animal cells, there has been increasing interest to study the biological properties of these algae polysaccharides within animal cells. An example for this are proteoglycans, which are found on the surface of animal cells and the extracellular matrix (ECM) and participate in structural and support functions. They have been shown to regulate a series of intercellular signaling pathways and interactions with cytokines and growth factors [2]. The structure of proteoglycans is similar to fucoidans, being composed of a protein (central chain) with glycosaminoglycans (GAGs) ramifications (e.g., chondroitin, dermatan, keratan, heparan sulphates, and heparin). This finding has sparked a renewed interest for studying the numerous potential biological properties including the anticoagulant [3], antioxidant [4], antiviral, immunomodulatory, anticomplement, and antitumor [5] characteristics of fucoidans isolated from different brown algae species.

The chemical variety of fucans in algae and invertebrate, their abundant bioavailability in nature as a renewable natural resource available from our coasts [5] and their potential use for biomedicine, make these polysaccharides an interesting material to study. This review will reveal not only structural characteristics but also the cellular/molecular aspects of fucoidans and their potential applications for cancer due to their properties to reduce metastasis and drug resistance in the different in vivo and in vitro cancer models.

## 2. General Structure of Fucoidans

Fucoidans from algae have been extensively studied since 1913 when Prof. Kylin discovered and described fucoidans [6]. Then, in 1957 these molecules were also shown to have anticoagulant functions and subsequently their anticancer activities were demonstrated (1970) [7].

**Figure 1:**
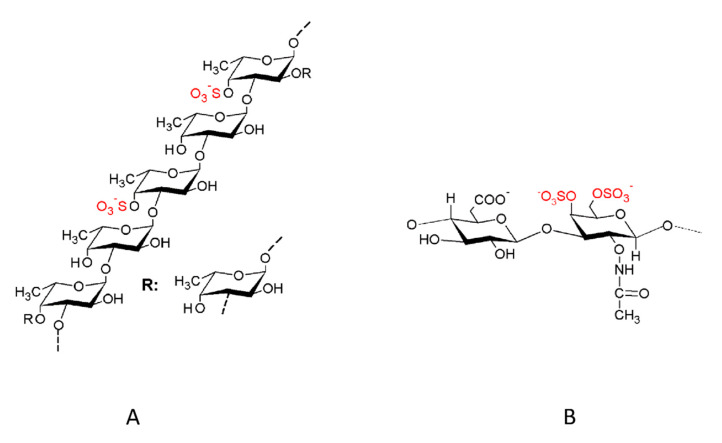
Comparison between fucoidan and glycosaminoglycan structures. (**A**) Structure of fucoidan from the brown alga *Fucus vesiculosus* and (**B**) structure of chondroitin sulphate. It is important to remark the similarity in the sugar skeleton and the presence of sulphate groups (red) in both structures. “R” represents a fucose subunit without sulphate.

As described above, fucoidans are polysaccharide composed by sulphated l-fucose (6-deoxy-l-galactose) [8]. Although many fucoidans consist of fucose and sulphate groups as is typical for fucans in general, fucoidans—in contrast to other fucans—consist of up to 10% of other monosaccharides (mannose, galactose, glucose, xylose, etc.), uronic acids, or branches of one or more monosaccharides [5]. In addition, there are fucoidans with different monosaccharide residues alternating with α (1→3) and α (1→4) bonds. Therefore, fucoidans constitute a highly variable and versatile subgroup of fucans [9] (Figure 1).

For instance, fucoidans from *Fucus vesiculosus* are composed of l-fucopyranosil residues linked through α (1→2) bonds with 4-position sulphate groups [10]. In addition, next generation techniques have shown that the scaffold is also composed by fucose residues linked through α (1→3) bonds with 4-position sulphate groups from some of the fucose residues disposed every two or three units of the main chain [11]. In contrast, other algae species contain the typical fucan complexes. *Sargassum stenophyllum* contains two types of fucans: (1) fucans containing predominantly α-l-fucose with high percentage of glucuronic acid and low amounts of sulphate located in different positions in the sugar [12] (2) fucans containing high amounts of sulphate but lower content of uronic acids distributed along the fucose chains or the only other sugar, galactose [12].

A wide range of l-fucose polymers has been found by fractionating the extracts from different algae species within the brown seaweed genus [3,13,14,15,16,17,18,19,20]. These fucoidans range from fractions of typical sulphated fucoidans to heteropolymer fractions of low-sulphate fucose and others containing glucosamine. The fucoidan structures vary from species to species, by season, location and maturity [21]. This structural variations are important for industrial applications to identify the optimum harvesting times and to ensure a consistent product composition. For instance, Fletcher et al., 2017 found that the highest quantity of fucoidans can be extracted from three algae *F. serratus*, *F. vesiculosus*, and *Ascophyllum nodosum* in autumn, whereas in spring the amount that can be obtained is at a minimum [21].

In addition to brown seaweed species, also marine invertebrates contain this type of sulphated polysaccharides. The viscous liquid containing sea urchin eggs, such as that of the *Strongylocentrotus franciscanus* species, contains a compound composed by sulphate acids residues only in position 2 bonds through α (1→3) bonds [22]. Other fucoidans have been found in the skin of the sea cucumber species *Stichopus japonicas* [23] and the recently commercially important *Holothuria tubulosa* [24].

The great diversity of fucoidans and their capability to be chemically modified make them molecules with great potential to be used as adjuvant agents in the treatment of cancer.

## 3. Fucoidans and Metastasis

In cancer, many cells develop the ability to invade adjacent tissue components of its primary organ and spread to other organs [25]. This process is called metastasis and involves several steps including altered cellular adhesions, cell motility, resistance to extracellular death signals, and disruption of the basement membrane and ECM [26]. Metastasis is responsible for more than 90% of cancer deaths [27] due to its systemic nature and higher drug resistance. Therefore, new molecular or clinical strategies are needed to counteract this aggressive feature [28]. In general, the metastasis process can be divided into 4 steps: (1) Certain tumor cells obtain characteristics of epithelial–mesenchymal transition (EMT), dissociating and detaching from the primary tumor to escapes from this area. (2) The dissociated tumor cells infiltrate into the surrounding stroma and invade and migrate through the basement membrane supporting the endothelium of local blood and/or lymphatic vessels. (3) The dissociated tumor cells cross the ECM resulting in intravasation. This involves dissemination of tumor cells to distant organs through blood or lymph vessels. These tumor cells can then forma new tumor in other organs or tissues (secondary tumor) through mesenchymal to epithelial transition (MET), which is another mechanism that enables metastatic colonization (neoplasm) and that is the contrary to EMT (e.g., re-expression of E-cadherin). (4) The final dormancy step is characterized by invading tumor cells that can remain silent for many years in the distant organ [29]. Both step 1 (EMT) and 2 (infiltration and invasion into stroma) are characterized by morphological changes from the epithelial cell monolayer with an apical-basal polarity, to dispersed, spindle-shaped mesenchymal cells with migratory protrusions [30]. In particular, EMT involves changes in the expression of cell–cell junction proteins, cytokeratin intermediate filaments, increase vimentin filaments and fibronectin [31]. In this case, sulphated fucoidans have been shown to maintain the endothelium adhesion by binding to endothelial cell receptors, especially when the polysaccharides that normally bind to these receptors decrease, confirming that fucoidans have antimetastatic effects and can prevent EMT [32]. A recent study demonstrated this using fucoidan from *F. vesiculosus*, which was able to inhibit the EMT and, therefore, an important step in the metastasis development [33]. In addition, fucoidan has been shown to decrease the activity or expression of transforming growth factor receptors (TGFRs) in vitro and in vivo. This blocks the EMT process and its morphological changes by upregulating epithelial markers, downregulating mesenchymal markers and decreasing the expression of transcriptional repressors such as SNAIL, SLUG, and TWIST, which subsequently induce migration and invasion inhibition [34]. Moreover, fucoidans are also able to reduce TGFR downstream signaling events, including SMAD2/3 and non-SMAD pathways: AKT, ERK1/2, and Focal Adhesion Kinase (FAK) phosphorylation. Fucoidans decrease TGFR proteins by ubiquitination proteasome pathway (UPP)-mediated degradation of TGFRs and by the promotion of SMURF2 and SMAD7 that conjugate to TGFRs, resulting in TGFR degradation [35].

Post-transcriptional mechanisms have also been implicated in the control of EMT and their relationship to TGF-β signaling through microRNAs (miRs). In this context, fucoidan of *S. hemiphyllum*, increases the miR-29 family expression that suppresses *DNMT3B* expression, which results in the upregulation of the tumor suppressor gene *MTSS1*. This fucoidan also downregulates TGF-β signaling, increases E-cadherin expression, decreases N-cadherin, *ADAM12*, and *PTEN* expression, and finally prevents ECM degradation by overexpressing *TIMP-1* and reducing the expression of matrix metalloproteinase enzymes MMP2 and MMP9, secreted by cancer cells to degrade ECM and induce cell migration [36,37]. Furthermore, an oligo-fucoidan extracted from *S. hemiphyllum* has been shown to inhibit the signaling of chemokine CCL2, which has a chemoattractant activity for monocytes, T cells, mast cells and basophils, and promotes invasion and metastasis via JAK-STAT and MAPK signaling pathways. Therefore, this CCL2 inhibition induces an inflammatory response, anti-tumor immunity and tissue conservation to avoid metastasis and angiogenesis [18]. Another example is the fucoidan of *S. fusiforme* which has an antimetastatic effect on liver cancer cells by inactivating the integrin αVβ3 and prevent the invadopodia formation [38].

Another characteristic of metastasis is the involvement of cell migration and invasion properties through ECM [30]. As fucoidans have structural similarities with heparin, these polysaccharides not only have anticoagulant features but also are able to decrease the expression and activity of matrix metalloproteinases, resulting in an incapability of tumor cells to cross the capillary wall [39]. For example, fucoidan derived from *Undaria pinnatifida sporophylls* inhibits in vitro cell growth, migration, invasion, and adhesion capabilities probably by downregulating the VEGFC/VEGFR3 axis, inactivating the NF-kB pathway and increasing the protein levels of TIMPs [40]. Other fucoidans decrease the expression levels of MMP2 in a dose dependent manner and downregulate the PI3K/Akt/mTOR signaling pathway [41].

Fucoidan of *Laminaria japonica* reduce the migratory and invasive features of triple-negative breast cancer (TNBC) cell models by suppressing the activation of MAPK and PI3K pathways and subsequently inhibiting AP-1 and NF-κB signaling. Additionally, this fucoidan was shown to inhibit micrometastasis in an in vivo transgenic zebrafish model [42].

**Figure 2:**
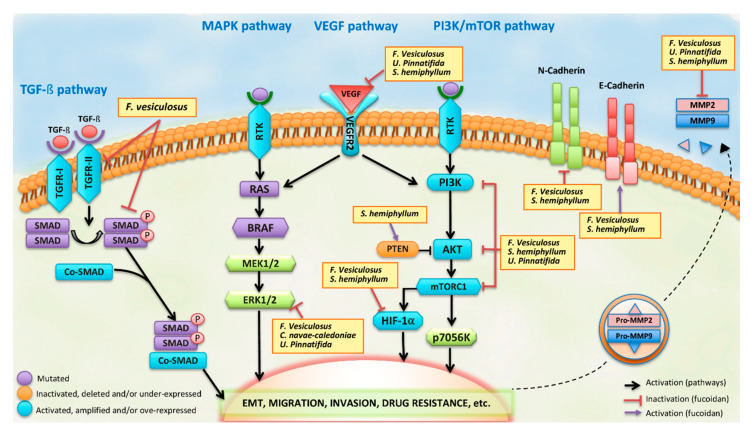
Summary of the main signaling pathways involved in the fucoidan function during the processes of metastasis and drug resistance.

Hypoxia in tumoral microenvironment is another phenomenon that can lead to metastasis. Fucoidan derived from *U. pinnatifida sporophylls* inhibit hypoxia in cancer cells through nuclear translocation, activity of HIF-1α and reduction in the levels of phosphorylated-PI3K (p-PI3K), p-Akt, p-mTOR, p-ERK, NF-κB, MMP-2, and MMP-9, but increased TIMP-1 levels. In addition, this fucoidan can decrease the levels of VEGF-C and HGF [43]. The most complete studies about inhibition of metastasis and drug resistance by fucoidans are shown in Table 1 and the main signaling pathways involved in these processes are shown in Figure 2.

Given the biological activities and implications of fucoidans in cancer, particularly in metastasis, the sulphated polysaccharides are candidates to generate functional foods and drugs as well as for their applications in prevention, synergism with chemotherapy, and nanotechnology. For instance, one nanotechnology application is the utilization of polysaccharides by eco-friendly synthesis of fucoidan-stabilized gold nanoparticles for charge interaction [44]. This demonstrates the potential of fucoidan to be used as a therapeutic agent and as technological material.

## 4. Fucoidans and Drug Resistance in Cancer

There are many types of cancer treatments, including surgery, radiation, chemotherapy, hormone therapy and, more recently, target therapy (e.g., chemokine receptors), stem cells transplantation, and immunotherapy [67]. One of the major complications in cancer treatment is the appearance of chemotherapy resistance, which is defined as the development of innate and/or acquired ability by cancer cells to evade the effects of chemotherapeutics [68]. Some cancer cells are intrinsically resistant to chemotherapy and others are able to develop a resistance phenotype, either by their own characteristics as tumor cells or by external conditions such as the tumor microenvironment [69]. For instance, repeated chemotherapeutic stimulation can induce pro-survival biological changes in tumor cells, allowing them to evade cell death under drug pressure by using host or tumor-related factors [70]. Most chemotherapeutic agents in cancer therapy (e.g., platinum drugs, taxanes) induce cell stress on “sensitive cells” resulting in cell death mediated predominantly by the apoptosis pathway [71]. Despite the effectiveness of programmed cell death induced by drugs, because tumors are heterogeneous in nature, certain cancer cells can display a drug-resistant behavior. This constitutes the main obstacle for anticancer therapeutic success [72]. There are four major mechanisms that contribute to drug resistance in cancer cells: (1) Decreased uptake of water soluble drugs [73]; (2) changes in intracellular pathways that affect the potential of cytotoxic drugs to kill cells, including alterations in the cell cycle, DNA repair, apoptosis pathways, metabolism/elimination of drugs, or others [73,74,75]; (3) increased energy-dependent efflux of hydrophobic drugs mediated via overexpression of a family of energy-dependent transporters (known as ATP-binding cassette transporters) such as P-glycoprotein 1 (P-gp, ABCB1) or breast cancer resistance protein (ABCG2) amongst others [73]; and (4) intracellular detoxifiers such as antioxidants (e.g., glutathione) [76,77]. Multiple signaling pathways have been implicated in resistance to chemotherapy, and innovative therapeutic strategies to overcome these are urgently needed [78].

Some fucoidans have been implicated in the decrease of the cancer drug resistant phenotype (Table 1). For example, fucoidans from *A. nodosum* showed an arrest in G1 phase of the cell cycle and a reduction in the chemoresistance to cisplatin of non-small-cell human bronchopulmonary carcinoma (NSCLC-N6) cells, a type of chemoresistant cell line [62]. The same study also showed an antitumor effect at sub-toxic doses of fucoidan in vivo in NSCLC-bearing nude mice [62]. Similarly, a sulphated fucan-like polysaccharide with aminosugar obtained from *Turbinaria ornate* was shown to arrest cell cycle in G1 phase in NSCLC-N6 cells [63]. A fucoidan obtained from *F. veciculosus* was able to decrease the expression of cellular prion protein (PrPC) HT29 colon cancer cell lines. PrPC is a protein whose overexpression is involved in increasing cell survival and proliferation, and inhibition of stress-response proteins p38, JNK, and p53, which could induce drug resistance [54,79].

More recently, cytokines have been shown not only to directly influence cancer progression by inducing cancer cell proliferation, migration, metastasis, reprogramming of tumor microenvironment (TME), immune evasion and the formation of new blood vessel within tumors [80,81] but are also often associated with chemoresistance and overall poor prognosis [80,82,83,84,85,86]. In this context, certain oligo-fucoidan have been shown to produce pro-inflammatory cytokines and chemokines (e.g., IL-6 and CCL2/MCP-1 respectively) and decrease the side effects of chemotherapy [18]. Also, other fucoidans can downregulate some cytokines and chemokines (e.g., M2-type chemokine CCL22) to inhibit tumor cell migration and lymphocytes recruitment via NF-κB-dependent transcription, which may be a novel and promising mechanism for tumor immunotherapy [46].

Fucoidans can also function as adjuvant agents along with chemotherapy. For instance, it has been demonstrated that sulphated polysaccharides can increase the bioavailability of certain oral drugs, like doxorubicin [87]. Fucoidans from *U. pinnatifida* and *F. vesiculosus* have been studied in combination with tamoxifen and paclitaxel in orthotopic mouse models of breast cancer and ovarian cancer. The results showed that both fucoidans improved the effect of tamoxifen, but not paclitaxel, in breast cancer. In the ovarian cancer model, only fucoidan from *F. vesiculosus* was able to improve the activity of tamoxifen, but not paclitaxel [50]. Fucoidan from *F. vesiculosus* has been shown to increase cytotoxicity of cisplatin on lung cancer cell lines via upregulation of cleaved caspase-3 and poly (ADP ribose) polymerase (PARP) expression, which induces apoptosis in these cells [47]. In addition, this fucoidan can also act synergistically with gefitinib to induce apoptosis in lung cancer cells [48].

Fucoidan from *U. pinnatifida* has also been investigated in melanoma, which is an intrinsically aggressive and therapy-resistant cancer that can develop resistance to the ERBB inhibitor, lapatinib. While, lapatinib alone inhibited 60% of tumor growth, in combination with fucoidan it decreased 85% of tumor growth. In addition, the use of fucoidan can counteract the morbidity associated with prolonged lapatinib treatment. This ability to avoid side effects provides an additional advantage for the potential use of fucoidan extracts [59]. Another fucoidan extracted from *Cladosiphon navae-caledoniae* Kylin in combination with cisplatin, tamoxifen or paclitaxel can improve outcomes in breast cancer treatment. These co-treatments significantly inhibited cell growth in MDA-MB-231 and MCF-7 breast cancer cells. Furthermore, they enhanced apoptosis in these cells by downregulating anti-apoptotic proteins Bcl-xL and Mcl-1 and promoting higher intracellular ROS levels [58].

Fucoidans have particular chemical characteristics (backbone with fucose sugar and sulphate group) that confer them a negative surface and favor interaction with other chemical compounds or cellular molecules. This makes them an interesting material for the development of nanoparticles. Hwang et al. designed fucoidan-cisplatin nanoparticles with high cisplatin content and loading efficiency. These were used to treat macrophage cells (RAW264.7) to assess immune protection from the cytotoxicity of cisplatin [88]. Indeed, the cells treated with fucoidan-cisplatin conjugation were more protected in comparison to cells treated with cisplatin alone. Moreover, the fucoidan-cisplatin nanoparticles showed stronger cytotoxicity against colon cancer cell lines than those treated with cisplatin alone, which suggests that fucoidan-based nanoparticles with high drug encapsulation have a potential application in immunotherapy and chemotherapy [88]. Other nanoparticles with fucoidan-coated manganese dioxide were applied in pancreatic cancer cell models associated to hypoxia as a mechanism of resistance to radiation therapy [56]. The nanoparticles not only showed a significant decrease of HIF-1 expression under a hypoxic condition, but they were also able to reverse hypoxia-induced radioresistance. The latter was shown by a decrease of clonogenic survival and an increase of DNA damage and apoptosis in response to radiation therapy. In vivo studies showed that fucoidan-coated manganese dioxide nanoparticles along with radiotherapy also decrease tumor growth in comparison to radiation alone [56]. Therefore, fucoidan-coated manganese dioxide nanoparticles have clinical potential in the treatment of hypoxic, radioresistant pancreatic cancer [56] (Figure 2). Furthermore, a combinational synergistic effect between fucoidan (natural compound), doxorubicin (chemotherapeutic drug) and photothermal nanocarrier (Pt nanoparticle) has been observed as it was possible to reverse the drug resistance of breast cancer cells submitted to photothermal therapy [66]. In this case, the fucoidan was applied as a biocompatible surfactant and surface-coating biopolymer in the fucoidan-coated photothermal nanocarrier. As a result, the biological–chemo–thermo combination treatment showed a promising therapeutic efficiency against multidrug resistant breast cancer cell MCF-7 ADR both in in vitro and in vivo breast cancer models [66]. Fucoidan from *F. vesiculosus* assembled within nanoparticles bearing doxorubicin improved significantly the chemotherapy response in breast cancer cell lines by enhancing their immunostimulatory activity [51].

The molecular mechanisms of drug resistance have been classified into pre-target (alterations that precede the binding to DNA), on-target (alterations that are directly related to drug-DNA interaction), post-target (mechanisms downstream of DNA damage with effect in cell death signaling pathways) and off-target (influencing on molecular processes that are not directly associated with drug-elicited signals) [78]. In this context, the potential mechanisms in which fucoidans can reverse the drug resistance are versatile. Fucoidans can inhibit chemokine/chemokine receptors interaction as a pre-target mechanism [18]. The increase of cell cytotoxicity and arrest of the cell cycle demonstrates their effect on on-target mechanisms [62].They can influence post-target mechanisms, for example through the downregulation of anti-apoptotic proteins Bcl-xL and Mcl-1.and finally, the promotion of higher intracellular ROS levels, is an example for their role in an off-target mechanisms [58].

## 5. Fucoidan Clinical Trials

In general, clinical trials are used to assess if a new treatment is more effective and/or has less harmful side effects than the standard treatment. Currently, only few clinical trials have been performed to assess fucoidan in cancer. These studies tested fucoidan either as a new therapeutic agent or as diet supplement (Table 2).

There are some examples of the use of fucoidan as a complementary therapy or food supplement in complementary alternative medicine in the treatment of cancer. A review, combining five case studies, showed clinical improvement in cancer patients, mainly using low molecular weight fucoidan supplements [93]. Other clinical trials in colorectal cancer [60] and breast cancer [89] in which fucoidans were used as a co-adjuvant treatment showed a better life quality cancer survivors [91] and in patients with advanced cancers [92]. The main fucoidan effects reported in cancer patients have been the improvement of negative effects of the chemotherapy and improved immune regulation. The fucoidan from *Cladosiphon okamuranus* for instance, decreases the cytotoxic effect from long-term colon cancer therapy (FOLFOX and FOLFIRI). The fucoidan in this case prevents the occurrence of fatigue during chemotherapy and increases patient survival. By ameliorating side effects, it enables the constant application of therapeutic drugs [90]. Fucoidan therefore has high potential for adjuvant therapy and may improve current clinical outcomes for cancer patients [55]. However, more clinical trials and further development of fucoidan applications are required.

## 6. Concluding Remarks

Fucoidans are a family of sulphated polysaccharides with great diversity in their structures due to their different sulphation patterns and the types of monosaccharides that in addition to fucose make up their backbone.

In some cancer types, fucoidans can inhibit metastasis processes including EMT, migration, invasion and MET processes. Fucoidans function by altering signaling axes such as TGFR/TGF-β, PI3K/AKT, VEGF, NF-κB, or ERK1/2 pathways and by inhibiting MMPs from cancer cells. Other mechanisms in which fucoidans may prevent EMT are TGF-β inhibition regulation of microRNAs. However, many questions regarding the functional mechanisms in which fucoidans affect EMT remain, leaving the door open for future research.

The molecular characteristics of fucoidans (e.g., molecular weight and sulphation grade) enable chemical or enzymatic modifications, which make them good candidates for therapeutic use, or to use them as adjuvants to increase the therapeutic efficiency of known chemotherapeutics. Moreover, the molecular versatility of fucoidans has made them excellent precursors for the development nanoparticles. Studies have demonstrated their potential to improve the efficiency of drug delivery into the tumor and/or to achieve a synergistic effect with other cancer drugs.

However, despite these auspicious/promising results, there is a lack of information about fucoidan structure, molecular weight, sulphate amount, etc. This will be important to better understand the possible influence of fucoidans on intracellular biological activity. In addition, the use of fucoidans in different cancer models and the interpretation of the results remains challenging. Most of the time, there are controversies related to the vague establishment of the studying variables or the scarce explanation of them, which makes it difficult to compare different studies.

Although there are still multiple challenges to overcome before fucoidans can be clinically used, it is predicted that in the near future, fucoidan-based approaches may provide important advances in overcoming the most complicated cancer drawbacks including metastasis and drug resistance and improving chemotherapy response and quality of life in cancer patients. Further studies are needed to discover more fucoidans and fucoidan-related targets to acquire a better understanding of how these molecules can arrest the mechanisms of metastasis and multidrug resistance in different cancer types.

## Figures and Tables

**Table 1:** Sources, characteristics and effects of fucoidans on the metastatic and drug-resistant phenotype of cancer models.

Source	Fucoidan Structure	Cancer Type/Model	Effects/Pathways	Refs
***Fucus vesiculosus***	This fucoidan has a central core formed by α-L-fucose (1,3)-linked, sulphated at C4. In addition, several branching points (every two or three fucose residues) were present in α-(1,2) or α-(1,4)-linked, on the main chain.	**Hepatocellular carcinoma (HCC)**		[45]
		In vitro Huh-7 and SNU-761 cell lines	Effects on metastasis by avoiding invasion ↑p42/44MAPK-dependent NDRG-1/CAP43 ↑p42/44 MAPK-dependent VMP-1	
		In vivo Distant metastasis model in C3H mice	Effects on metastasis by avoiding invasion ↓MMPs (MMP-2) ↓NF-κB ↓VEGF	
		In vitro MHCC-97H cell line	Nanoparticle drug resistance fucoidan downregulate chemokines and cytokines involved in chemoresistance	[46]
		**Lung cancer**		[35]
		In vitro NSCLC CL1-5 human cells A549 human cells LLC1 mouse cells	Effects on metastasis by avoiding migration and proliferation ↓TGFRI and TGFRII ↓p-SMAD2/3 ↓AKT ↓ERK1/2 ↓p-FAK	
		In vivo Xenograft	Drug resistance and Combined therapy ↑ Cisplatin cytotoxicity ↑Caspase 3, PARP and apoptosis	[47]
		Lung cancer cell line *In vitro*	Synergize with gefitinib and ↑apoptosis	[48]
		**Breast cancer**		[34]
		In vitro MDA-MB-231 and MCF-7 human breast cancer cells In vivo 4T1 mouse breast adenocarcinoma	Effects on metastasis by avoiding EMT ↑E-Cadherin, ↑γ-Catenin ↓N-Cadherin ↓SNAIL, SLUG and TWIST ↓p-SMAD2/3 ↓SMAD4 ↓TGFRI and TGFRII ↓MMP-9	
		In vitro MDA-MB-231 cells	Effects on metastasis by avoiding EMT ↓N-Cadherin and ↓vimentin ↑ZO-1, ↑E-Cadherin ↓Nuclear translocation of HIF-1α ↓TWIST-1, SNAIL, CAIX and GLUT-1	[49]
		In vitro MCF-7 and ZR-75 In vivo Orthotopic Mouse model	Combined therapy increase effect Tamoxifen	[50]
		In vitro MDA-MB-231 human breast cancer cells, 4T1 mouse breast cells and J774.1A mouse macrophage cells. In vivo BALB/c mice	Nanoparticle combined therapy ↑ immunostimulatory activity and increase doxorubicin effect	[51]
		**Proliferative vitreoretinopathy (PVR)**		[52]
		In vitro Human primary RPE cells In vivo PVR model in rabbits	Effects on metastasis by avoiding EMT ↓TGF-β1-induced SMAD2/3 phosphorylation ↓α-SMA and fibronectin ↓E-cadherin	
		**Colorectal cancer (CRC)**		[41,53]
		In vitro HT29 human cells	Effects on proliferation ↓Cyclin D1/E and ↓CDK2/4 Effects on apoptosis ↓BCL2 ↑BAX, ↑Caspase-3, ↑PARP1	
		In vitro HT29 human colon cancer cells	Effects on metastasis by avoiding migration ↓MMP-2 ↓PI3K-AKT-mTOR drug resistance by effect in P38 and JNK pathways	[41]
			Drug resistance related decrease prion protein and decrease cell survival and could	[54]
		HCT-8 human ileocecal In vitro	Combined therapy ↑cytotoxicity than those treated with cisplatin alone	[55]
		**Pancreatic cancer**		[56]
		In vitro AsPC-3 and BxPC-3 human pancreatic cancer cell lines	Effects on metastasis by avoiding hypoxia and angiogenesis ↓Hypoxia induced radioresistance ↓HIF-1α ↓Tumor growth and angiogenesis	
		In vivo Xenograft	Combined therapy	
		**Prostate cancer**		[57]
		In vitro DU-145 human cells In vivo Xenograft	Effects on metastasis by avoiding angiogenesis ↓CD31 and CD105 ↓p-JAK and p-STAT3 ↓VEGF, Bcl-xL, Cyclin D1	
***Cladosiphon navae-caledoniae***	Low molecular weight fraction (72%, MW < 500 Da) and non-digested fractions (less than 28%, peak MW: 800 kDa). Fucose (73%), xylose (12%) and mannose (7%). The ratio of sulphation was 14.5%.	**Breast cancer**		[58]
		In vitro ER-positive MCF-7 cells ER-negative MDA-MB-231 cells	Effects on metastasis and apoptosis ↓p-ERK and ↓AKT in MDA-MB-231 cells ↑p-ERK in MCF-7 cells ↑IC-ROS and ↓GSH in both cell lines	
			Effects on drug resistance ↑cisplatin, tamoxifen and paclitaxel efficacy ↓Cell growth, ↑apoptosis ↓Bcl-xL, ↓Mcl-1 ↑ROS Combined therapy	
***Undaria pinnatifida***	This sulphated galactofucan is composed of: Galactose 44.6% and Fucose 50.9%. Xylose (4.2%) Mannose (0.3%), uronic acids were not detected. A significant number of O-acetyl groups	**Hepatocellular carcinoma (HCC)**		[20,40,43]
		In vitro Hca-F cell line	Effects on metastasis ↓VEGF C/VEGFR 3 ↓HGF/c-MET, cyclin D1. ↓PI3K, p-AKT, p-ERK 1/2, and NF-κB Effects on metastasis by avoiding hypoxia ↓HIF-1α ↓p-PI3K, ↓p-AKT, ↓p-mTOR ↓p-ERK ↓NF-κB ↓MMP-2, ↓MMP-9 ↑TIMP-1	
		In vivo Hca-F cells were inoculated subcutaneously into the footpads of the mice	Effects on metastasis by deregulating adhesion/invasion ↓ L-Selectin ↑TIMPs Effects on metastasis by avoiding lymph angiogenesis and lymphatic infiltration ↓VEGF-C, ↓HGF	
		**Melanoma cancer**		
		In vitro WM266-4, WM115 (mutated BRAF), SKMEL2 (RAS mutated), MeWo and FEMX (wild type)	Effects on drug resistance and combined therapy Fucoidan increase Lapatinib (ERBB inhibitor) effect in drug resistance cell	[59]
		**Breast cancer**		
		In vitro MCF-7 and ZR-75 In vivo Orthotopic Mouse model	Combined therapy Increase effect in Tamoxifen treatment	[50]
***Sargassum hemiphyllum***		**Colorectal cancer (CRC)**		[60]
		Double-Blind Randomized Controlled Trial	Fucoidan as a supplemental therapy to chemotarget agents in patients with metastatic CRC	
		**Hepatocellular carcinoma (HCC)**		[36]
		In vitro Huh6, Huh7, SK-Hep1 and HepG2 human cells.	Effects on metastasis by avoiding EMT ↑miR-29b, ↓DNMT3B, ↑MTSS1 ↑E-Cadherin, ↓N-Cadherin	
			↑TIMP-1, ↓MMP-2/9	
		**Breast Cancer**		[37]
		In vitro MCF-10A, MCF-7	Effects on metastasis by avoiding migration and invasion ↑miR-29c, ↓ADAM12 ↓miR-17-5p, ↑PTEN	
		MDA-MB-231 human cells.	Effects on metastasis by avoiding EMT ↑E-Cadherin, ↓N-Cadherin	
***Ascophyllum nodosum***	This fucoidan is composed of fucose (52.1%), galactose (6.1%), glucose (21.3%), and xylose (16.5%). Sulphate content is 19%. Two main size fractions (47 and 420 kDa).	**NSCLC (Lung cancer)**		[61,62]
		In vitro NSCLC-N6	Effects on cell cycle arrest	
		In vivo Xenograft		
***Turbinaria ornate***	The results showed that the fucoidan has a sulphate content of 25.6% and is mainly composed of fucose and galactose residues (Fuc:Gal ≈ 3:1). The fucoidan has a backbone of 3-linked α-L-Fucose residues with branches, →4)-Galp(1→ at C-4 of the fucan chain. Sulphate groups are attached mostly at C-2 and sometimes at C-4 of both fucose and galactose residues.	**NSCLC (Lung cancer)**		[63,64]
		In vitro NSCLC-N6	Effects on cell cycle arrest	
***Cladosiphon okamuranus***	The fucoidan is composed of 70.13 ± 0.22 wt% fucose and 15.16 ± 1.17 wt% sulphate. Other minor monosaccharides are D-xylose, D-galactose, D-mannose, D-glucose, D-arabinose, D-rhamnose and D-glucuronic acid. Linkage analysis revealed that fucopyranoside units along the backbone are linked, through α-1,3-glycosidic bonds, with fucose branching at C-2, and one sulphate group at C-4 per every three fucose units, i.e. the structure of fucoidan from Japanese Cladosiphon okamuranus is [→3)-α-fuc(1→]0.52[→3)-α-fuc-4-OSO3-(1→]0.33[→2)-α-fuc]0.14.	**Breast cancer**		[65,66]
		In vitro MCF-7 ADR (drug resistant human breast cancer cell line)	Combination therapy (Synergistic effect doxorubicin and photothermal nanocarrier) ↑doxorubicin delivery ↑ morphology-control in Pt-nanoparticles	
		In vivo Xenograft		
***Sargassum fusiforme***	The fucoidan is composed of fucose, xylose, galactose, mannose, glucuronic acid, and 20.8% sulphate. The 17 sulphate groups are attached to diverse positions of fucose, xylose, mannose, and galactose residues. The backbone consists of alternate 1, 2-linked α-D-Mannose and 1, 4-linked β-D-GlcpA	**Hepatocellular carcinoma (HCC)**		[38]
		In vitro SMMC-7721, Huh7 and HCCLM3 cells	Effects on metastasis by avoiding migration and invasion	
		In vivo Xenograft	↓Invadopodia-related proteins (Src, Cortactin, N-WASP, ARP3, CDC42, MMP2, MT1-MMP) ↓Integrin αVβ3	

α-SMA: α-smooth muscle actin. CDK: Cyclin dependent kinase. CRC: Colorectal cancer. CTGF: Connective tissue growth factor. EMT. Epithelial-mesenchymal transition. ER: Estrogen receptor. FAK: Focal adhesion kinase. FE: Fucoidan extract. GSH: Glutathione. HCC: Hepatocellular carcinoma. HGF: hepatocyte growth factor. CRC: colorectal cancer. NSCLC: Non-small-cells human bronchopulmonary carcinoma. IC-ROS: Intra cellular reactive oxygen species. LMWF: Low molecular weight fucoidan. MMP: Matrix metalloproteinase. NDRG: N-myc downstream-regulated gene. PTEN: phosphatase and tensin homolog. PVR: Proliferative vitreoretinopathy. ROS: Oxygen reactive species. RPE: Retinal pigment epithelial. TGFR: Transforming growth factor-b receptor. TIMP: Tissue inhibitor of metalloproteinase. VEGF: Vascular endothelial growth factor. VMP: vacuole membrane protein.

**Table 2:** Fucoidans tested in clinical trials.

Source	Cancer Type (No Patients)	Fucoidan Dosage	Effects	Refs
*Undaria pinnatifida*	Breast cancer (20 patients)	Capsule of 500 mg twice a day for 3 weeks	Letrozole (n = 10) or Tamoxifen (n = 10) co-administration with fucoidan no decrease drugs in steady-state plasma and was well tolerated.	[89]
*Sargassum hemiphyllum*	Colorectal cancer (54 patients)	4 g twice a day for 6 months	Supplemental therapy, fucoidan combined with FOLFIRI chemotherapy plus Bevacizumab improved disease control rate.	[60]
*Cladosiphon okamuranus*	Unresectable advanced or recurrent cases of colorectal cancer (20 patients).	4.05 g for day	Decreases toxicity of chemotherapy FOLFOX or FOLFIRI.	[90]
	Survivors of diverse cancer types (11 patients).	1.5 g twice a day for 6 months	Activation of NK cells in male cancer survivors	[91]
	Advanced cases of several types of cancer (20 patients).	4 g for day for 4 weeks	Anti-inflammatory effect, decreases IL-1β, IL-6 and TNF-α	[92]
*Nemacystis decipiens*	Cervical cancer (1 case study) Kidney cancer (1 case study) Breast cancer (1 case study)	200 mL/day 60 mLx3L/day 200 mL/day	No concluded information	[93]

FOLFIRI: Combination chemotherapy with Irinotecan plus 5-Fuorouracil/leucovorin; FOLFOX: Combination chemotherapy with Oxaliplatin plus 5-Fuorouracil/leucovorin; NK: Natural Killer; IL-1β: Interleukin 1-β; IL-6: Interleukin 6; TNF-α: Tumor Necrosis Factor-α.

## Abbreviations

DNMT3B	DNA methyltransferase 3B
MTSS1	metastasis suppressor 1
ADAM12	a disintegrin and metalloproteinase 12
PTEN	phosphatase and tensin homolog
TGF-β	Transforming growth factor beta (β)
TGFRs	Transforming growth factor b receptors
VEGFC	vascular endothelial growth factor C
VEGFR3	VEGF receptor 3
TIMPs	tissue inhibitor of metalloproteinases
MMP	matrix metalloproteinase
NF-κB	nuclear factor kappa-beta
ECM	extracellular matrix
GAGs	glycosaminoglycans
EMT	epithelial-mesenchymal transition
FAK	Focal adhesion kinase
UPP	ubiquitination proteasome pathway
miRs	microRNAs
P-gp	P-glycoprotein 1
ABCB1	ATP Binding Cassette Subfamily B Member 1
ABCG2	breast cancer resistance protein
PrPC	cellular prion protein
TME	tumor microenvironment
IL-6	interleukin-6
CCL2/MCP-1	chemokine (C-C motif) ligand 2/ monocyte chemoattractant protein 1
ROS	reactive oxygen species
HIF-1	Hypoxia Inducible Factor
FOLFIRI	Combination chemotherapy with Irinotecan plus 5-Fuorouracil/leucovorin
FOLFOX	Combination chemotherapy with Oxaliplatin plus 5-Fuorouracil/leucovorin
